# L-Proline supplementation preserves sperm function and chromatin integrity in asthenozoospermic patients during cryopreservation

**DOI:** 10.1186/s12610-025-00286-x

**Published:** 2025-10-13

**Authors:** Mojtaba Moradi, Masoumeh Golestan Jahromi, Elham Ghanbari, Amir Hossein Hashemian, Azita Faramarzi

**Affiliations:** 1 Fertility and Infertility Research Center, Health Technology Institute, Kermanshah University of Medical Sciences, Kermanshah, Iran; 2 Department of Advanced Medical Sciences & Technologies, School of Medicine, Jahrom University of Medical Sciences, Jahrom, Iran; 3 Department of Tissue Engineering, School of Medicine, Kermanshah University of Medical Sciences, Kermanshah, Iran; 4 Department of Biostatistics, School of Health, Kermanshah University of Medical Sciences, Kermanshah, Iran; 5 Research Center for Environmental Determinants of Health (RCEDH), Health Institute, Kermanshah University of Medical Sciences, Kermanshah, Iran; 6 Department of Anatomical Sciences, Medical School, Kermanshah University of Medical Sciences, Kermanshah, Iran

**Keywords:** Proline, Semen analysis, Asthenozoospermia, Chromatin, Oxidative stress, Antioxidants, Proline, Analyse du Sperme, Asthénozoospermie, Chromatine, Stress oxydatif, Antioxydants

## Abstract

**Background:**

Asthenozoospermia is a major cause of male infertility, accounting for approximately 18% of infertility cases. L-Proline, a natural antioxidant and osmoprotectant, has gained attention for its potential applications in semen cryopreservation. This study aimed to evaluate the beneficial effects of L-proline on sperm quality in samples from asthenozoospermic patients during cryopreservation.

**Results:**

This in vitro experimental study utilized semen samples from 30 men (aged 27–40 years) diagnosed with asthenozoospermia. Each sample was divided into three aliquots for cryopreservation: (1) a control group using a standard unsupplemented freezing medium; (2) a group supplemented with 2 mM L-proline; and (3) a group supplemented with 4 mM L-proline. Following thawing, samples were assessed for sperm count, motility, viability, morphology, and chromatin quality. Additionally, levels of malondialdehyde (MDA), total antioxidant capacity (TAC), and nitric oxide (NO) were assessed. Supplementation with 2 mM L-proline resulted in significantly higher post-thaw progressive motility, viability, and chromatin integrity compared to the control group (*p* < 0.05). These functional improvements were associated with a more favorable antioxidant status, evidenced by significantly lower levels of MDA and NO and higher levels of TAC (*p* < 0.05). While the 4 mM L-proline group showed some protection, the effects were less pronounced than those observed with the 2 mM concentration.

**Conclusions:**

The results suggest that adding 2 mM L-proline into the freezing medium effectively protects human sperm quality and chromatin integrity in asthenozoospermic samples by mitigating cryopreservation-induced nitro-oxidative stress. This strategy holds promise for improving assisted reproductive technology (ART) outcomes in men with asthenozoospermia.

## Résumé

**Contexte:**

L’asthénozoospermie est une cause majeure d’infertilité masculine, représentant environ 18 % des cas d’infertilité. La L-Proline, un antioxydant et osmoprotecteur naturel, a attiré l’attention pour ses applications potentielles dans la cryoconservation du sperme. Cette étude visait à évaluer les effets bénéfiques de la L-proline sur la qualité du sperme, dans des échantillons de patients asthénozoospermiques, lors de la cryoconservation.

**Résultats:**

Cette étude expérimentale *in vitro* a utilisé des échantillons de sperme de 30 hommes (âgés de 27 à 40 ans) diagnostiqués avec une asthénozoospermie. Chaque échantillon a été divisé en trois aliquotes pour la cryoconservation : (1) un groupe témoin utilisant un milieu de congélation standard non supplémenté ; (2) un groupe supplémenté avec 2 mmol/L de L-proline ; et (3) un groupe supplémenté avec 4 mmol/L de L-proline. Après décongélation, les échantillons ont été évalués pour le nombre, la mobilité, la viabilité, et la morphologie de spermatozoïdes, et sur la qualité de la chromatine. De plus, les niveaux de malondialdéhyde (MDA), de la capacité antioxydante totale (TAC), et d’oxyde nitrique (NO) ont été évalués. La supplémentation de 2 mmol/L de L-proline a entraîné une motilité progressive, une viabilité et une intégrité de la chromatine significativement plus élevées après décongélation par rapport au groupe témoin (*p* < 0,05). Ces améliorations fonctionnelles ont été associées à un statut antioxydant plus favorable, mis en évidence par des niveaux significativement plus faibles de MDA et de NO et des niveaux plus élevés de TAC (*p* < 0,05). Bien que le groupe de 4 mmol/L de L-proline ait montré une certaine protection, les effets étaient moins prononcés que ceux observés avec la concentration de 2 mmol/L.

**Conclusions:**

Les résultats suggèrent que l’ajout de 2 mmol/L de L-proline dans le milieu de congélation protège efficacement la qualité du sperme humain et l’intégrité de la chromatine, dans les échantillons asthénozoospermiques, en atténuant le stress nitro-oxydatif induit par la cryoconservation. Cette stratégie est prometteuse pour améliorer les résultats de la technique de procréation médicalement assistée chez les hommes atteints d’asthénozoospermie.

## Background

Male infertility contributes to nearly half of infertility cases globally, with a notable decline in sperm quality being a primary concern in global health [1, 2]. Asthenozoospermia, characterized by reduced sperm motility, is a significant factor in male infertility, impairing the ability of sperm to reach and fertilize the oocyte [3]. While sperm cryopreservation is a critical component of assisted reproductive technologies (ART) for fertility preservation, the freeze–thaw process induces considerable cellular stress, leading to declines in sperm quality and functionality [4]. This problem is especially severe in asthenozoospermic patients. Their spermatozoa are already compromised, and the cryopreservation process exacerbates this by intensifying cellular damage, disrupting membrane integrity, and ultimately reducing the chances of successful fertilization post-thaw [5].

Sperm cells are highly vulnerable to cryodamage, largely due to heightened oxidative and osmotic stress during the freeze–thaw process [6, 7]. This cycle disrupts the delicate balance between reactive oxygen species (ROS) and antioxidant defenses, resulting in excessive lipid peroxidation (LPO) [6, 7]. Consequently, the structural integrity of the sperm membrane is compromised, mitochondrial function is impaired, and DNA becomes prone to fragmentation [8]. Together, these cryopreservation-induced stresses markedly reduce post-thaw sperm motility and viability, posing a substantial challenge to the success of assisted reproductive technologies (ART) [9].

To counter this cryodamage, recent research has focused on supplementing the freezing medium with protective antioxidant agents [10, 11]. Among these, the nonessential amino acid L-proline (proline) has emerged as a particularly promising candidate due to its multifaceted roles in cellular protection. Proline acts as a potent antioxidant, neutralizing ROS to protect the sperm membrane and DNA from oxidative damage [12–14]. Recent evidence suggests that proline supplementation can enhance sperm function, as studies have shown improvements in motility, viability, and chromatin integrity [13]. Our previous studies have also shown that proline can effectively preserve human sperm quality during incubation and cryopreservation [15, 16]. Uniquely, it also functions as an osmoprotectant, helping to stabilize cellular volume and membrane integrity during the harsh osmotic shifts of freeze–thaw cycles. Additionally, it may serve as an energy substrate, enhancing sperm motility and longevity. Recent studies further suggest its involvement in modulating signaling pathways critical for sperm capacitation and the acrosome reaction [15, 17].

Despite these promising findings, the specific protective effects of proline on the particularly vulnerable sperm from asthenozoospermic men have not been characterized. Therefore, we hypothesized that supplementing the cryopreservation medium with proline would mitigate cryopreservation-induced damage in sperm from asthenozoospermic men by reducing nitro-oxidative stress. This study aims to test this hypothesis by investigating the effects of different proline concentrations on post-thaw sperm parameters, including progressive motility, viability, morphology, and chromatin integrity, as well as on biochemical markers of oxidative status (MDA, TAC, and NO) in asthenozoospermic samples.

## Materials and methods

### Chemicals

The chemicals used in the present study, including proline, were purchased from Sigma-Aldrich Co, unless otherwise noted.

### Semen collection and preparation

This is an in vitro study that was conducted between July 2023 and July 2024 and was approved by the Institutional Review Board of Kermanshah University of Medical Sciences, Iran (IR.KUMS.MED.REC.1401.109). It involved 30 healthy men, all partners of infertile couples, referred to the Andrology Division and IVF clinic at Motazedi Hospital, Kermanshah, Iran. Written informed consent was obtained from all participants prior to their inclusion. Semen samples were collected through masturbation after a period of 72 h of sexual abstinence. Sperm analysis was performed in accordance with World Health Organization (WHO, 2010) guidelines, assessing semen volume, sperm concentration, total motility, and morphology. Inclusion criteria required samples to meet asthenozoospermia parameters, defined as less than 40% total motility or less than 32% progressive motility, with a minimum semen volume of 1.5 ml, a sperm concentration of at least 15 million spermatozoa per ml, and ≥ 4% normal morphology. Participants completed a detailed questionnaire addressing their occupation, health status, smoking and alcohol consumption habits, diet, and exposure to environmental pollutants. Exclusion criteria included smoking, alcohol consumption, male accessory gland infections, systemic diseases, micro-orchidism, cryptorchidism, varicocele, or prior use of hormonal treatments, medications, vitamins, or antioxidants. Semen samples were prepared for analysis using a discontinuous double-layered density gradient centrifugation method, following established WHO protocols (WHO, 2010) [15, 18].

### Study design

To prepare the experimental treatments, proline (Sigma-Aldrich, Catalog. No. P0380) powder (1.15 mg) was dissolved in 1 mL of Ham’s F10 medium (Gena Teb, Cat. BI-1013) to create a stock solution. Working concentrations of 0, 2, and 4 mM were freshly prepared each day and incorporated into the cryopreservation medium. To ensure intra-sample consistency and minimize inter-individual variability, each semen sample was divided into three equal aliquots. Each aliquot was then cryopreserved using one of the three proline concentrations:

- Group 1 (control): 0 mM proline (no supplementation)
- Group 2: 2 mM proline
- Group 3: 4 mM proline

All aliquots underwent identical cryopreservation procedures. Sperm parameters were evaluated both before and after cryopreservation to determine the effects of proline supplementation on sperm quality [16].

### Cryopreservation and thawing

For cryopreservation, semen samples were diluted at a 1:1 ratio with a commercial freezing medium (Sperm Freeze Solution-10137, Vitrolife, Gothenburg, Sweden) at room temperature. The diluted samples were carefully transferred into cryotubes and equilibrated at room temperature for 10 min. After equilibration, the cryotubes were positioned 10–15 cm above liquid nitrogen (LN2) in nitrogen vapor for 15–20 min before being fully submerged in LN2 for storage. To thaw the samples, the cryotubes were placed in a water bath maintained at 35 ± 2 °C for 5 min. The thawed specimens were mixed with pre-warmed Ham's F10 medium and centrifuged to remove the cryoprotectant. Finally, the sperm pellets were resuspended in an appropriate volume of Ham's F10 medium for subsequent analyses [16].

### Evaluation of sperm parameters

Sperm count was determined using a Neubauer chamber and light microscopy (Olympus Co., Tokyo, Japan). Sperm viability was evaluated using the one-step eosin-nigrosin staining (Sigma-Aldrich) method. A minimum of 200 spermatozoa per sample were analyzed under phase-contrast microscopy at 1000 × magnification. Sperm with unstained heads were classified as viable, whereas those with dark pink or red-stained heads were considered non-viable. Sperm motility was analyzed by categorizing 200 spermatozoa into progressive or non-progressive motile groups, following the guidelines established by the World Health Organization (WHO, 2010) [19]. The percentage of motile sperm was determined using phase-contrast microscopy at 400 × magnification. Sperm morphology was assessed using the Papanicolaou staining method (BRED, Cat. No. BRED-031), adhering to WHO (2010) criteria. At least 200 spermatozoa per sample were examined under phase-contrast microscopy at 1000 × magnification (Moradi et al., 2021) [16, 20].

### Measurement of lipid peroxidation levels

MDA concentration was measured using the ZellBio MDA assay kit (Catalog ID: ZX-44116 −96, ZellBio GmbH, Germany). For the assay, 50 µl of reagent 4 was added to 50 µl of semen sample, followed by 1 µl of the prepared chromogen solution. The mixture was transferred to microtubes, which were then subjected to a boiling water bath followed by an ice bath for 1 h. After incubation, the microtubes were centrifuged at 3000–4000 × g for 10 min. The absorbance of the resulting supernatant was measured at 535 nm using an ELISA microplate reader [16].

### Total antioxidant capacity measurement

The TAC was assessed using the ZellBio TAC assay kit (CatalogID: ZX-44109 −192, ZX-44109 −96, ZellBio GmbH, Germany). For each sample, 10 µl of sperm sample or reconstituted Trolox at varying concentrations (used as standards) was added to the wells of a microplate. This was followed by the addition of 190 µl of the prepared working chromogen reagent. After a 2-min incubation at room temperature, the absorbance was measured at 490 nm using a plate ELISA reader [16].

### Assessment of nitric oxide levels

NO levels in sperm samples were measured using the Griess reagent assay. Following cryopreservation, sperm samples were diluted to a concentration of 5 × 10⁶ cells/ml in phosphate-buffered saline (PBS, pH 7.4) and aliquoted into sterile tubes. Samples were incubated in substrate buffer containing HEPES (25 mM), NaCl (140 mM), KCl (5.4 mM), CaCl₂ (1 mM), MgCl₂ (1 mM), and NADPH (1.44 mM) with nitrate reductase (20 mU) to convert nitrates to nitrites. The reaction was carried out at room temperature for 1 h and stopped by freezing and thawing the samples. After centrifugation at 1500 × g for 15 min, the supernatant was mixed with an equal volume of Griess reagent (1% sulfanilamide, 0.1% naphthylenediamine dihydrochloride, and 2.5% phosphoric acid). The formation of a colored azo dye was quantified by measuring absorbance at 543 nm using a spectrophotometer. Nitrite concentrations were calculated from a sodium nitrite standard curve and expressed as µmol NO per 10⁶ cells [20, 21].

### Evaluation of sperm chromatin dispersion

The sperm chromatin dispersion (SCD) assay was performed to evaluate chromatin integrity, relying on the principle that sperm with intact chromatin form a halo of dispersed chromatin loops after acid denaturation. In contrast, those with fragmented chromatin lack this halo. The assay was conducted following standardized protocols, ensuring reproducibility and accuracy. A minimum of 200 spermatozoa per sample were analyzed under phase-contrast microscopy (Olympus Co., Tokyo, Japan) at 400 × magnification. The presence or absence of the chromatin halo was used as an indicator of chromatin integrity, with a fragmented chromatin profile identified by the absence of a visible halo [15].

### Statistical analysis

Data normality was assessed using the Shapiro–Wilk and Kolmogorov–Smirnov tests. For group comparisons, one-Way ANOVA with Tukey's post-hoc test and the Friedman test were employed to evaluate differences in various parameters across groups. A *p*-value of less than 0.05 was regarded as statistically significant. All statistical analyses were conducted using SPSS software (Version 27).

## Results

### The effects of L-proline on sperm parameters

**Fig. 1:**
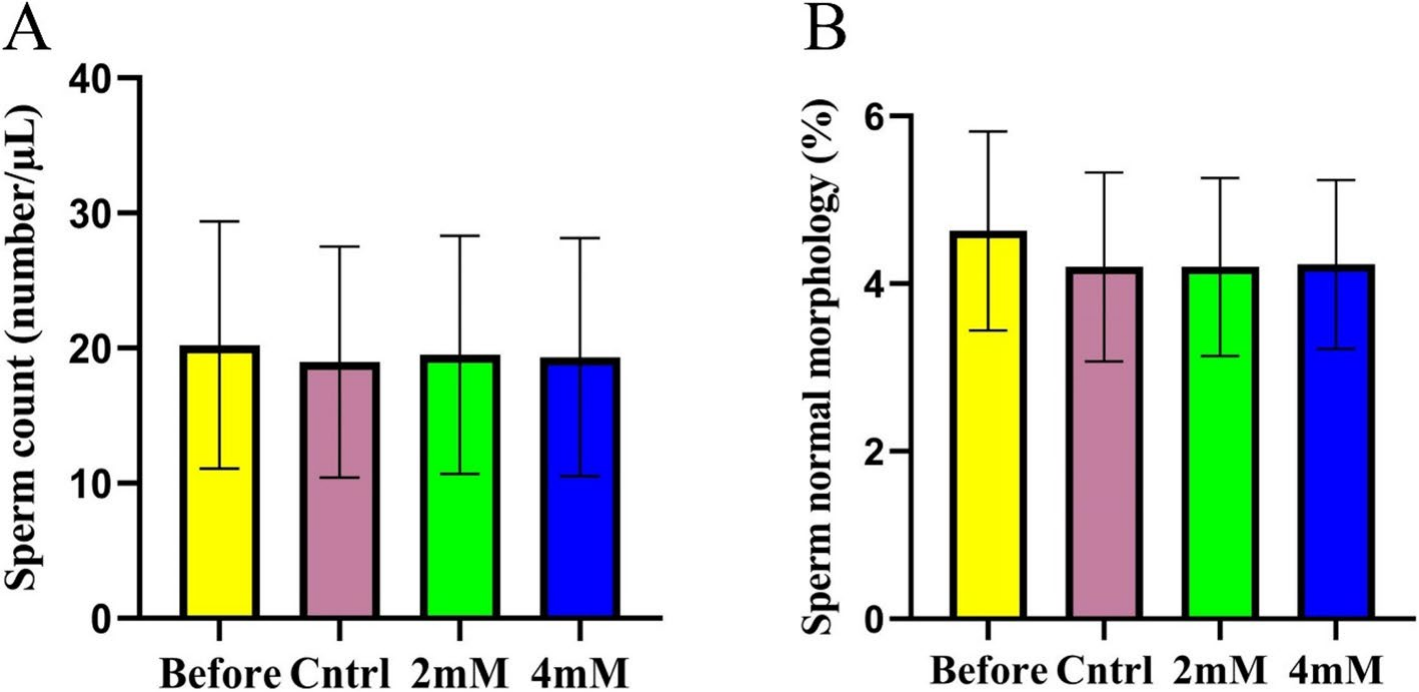
The effect of different concentrations of proline on the sperm count and sperm morphology during cryopreservation (*n* = 30), presented as mean ± SD. **a** *p* < 0.05 versus before cryopreservation; **b** *p* < 0.05 versus the control group;**c** *p* < 0.05 versus 2 mM group

As anticipated, the cryopreservation process did not notably change sperm count and morphology across the treatment groups. The addition of different concentrations of proline had no significant effect on sperm count (*p* > 0.05) (Effect size = −0.102) (Fig. 1-A). Likewise, the inclusion of different concentrations of proline in the freezing medium did not remarkably change the sperm morphology during cryopreservation (*p* > 0.05) (Effect size = −0.013) (Fig. 1-B).

**Fig. 2:**
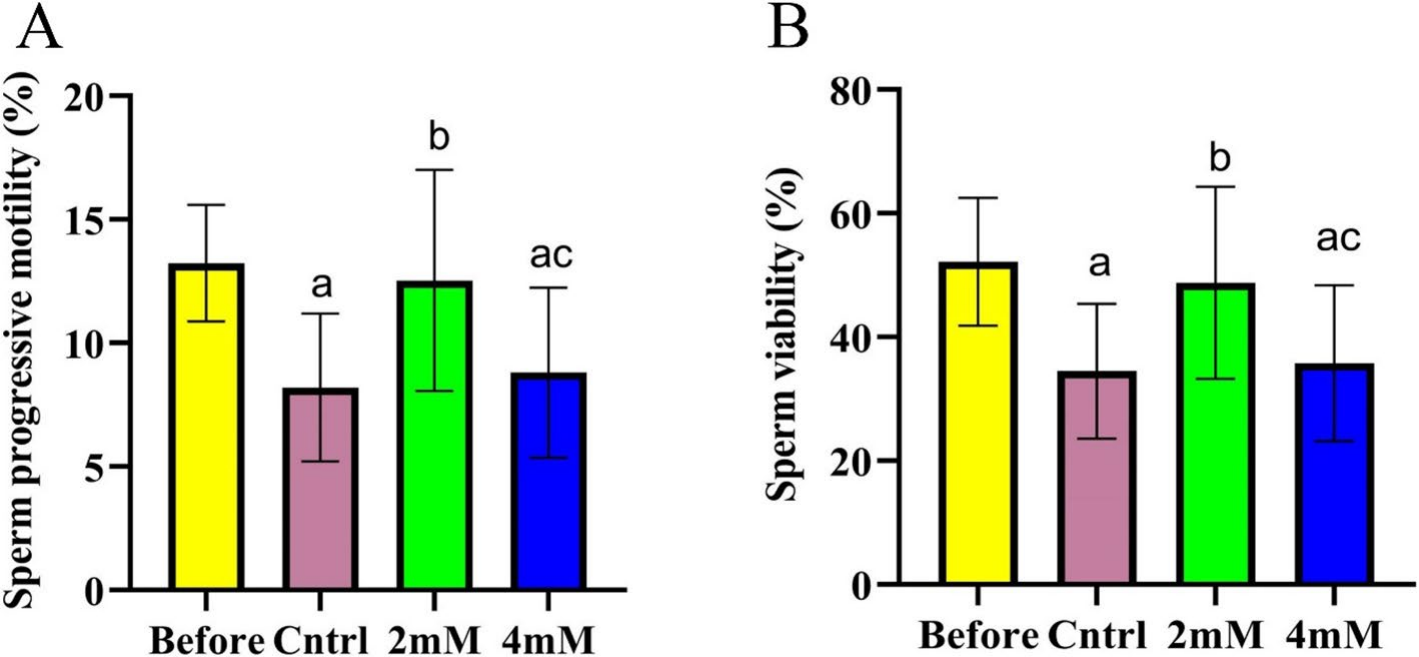
The effect of different concentrations of proline on the sperm progressive motility and sperm viability during cryopreservation (*n* = 30), presented as mean ± SD. **a** *p* < 0.05 versus before cryopreservation; **b** *p* < 0.05 versus the control group;**c** *p* < 0.05 versus 2 mM group

Additionally, our results indicate that the freezing–thawing process can negatively affect sperm progressive motility (*p* < 0.001). While the inclusion of 2 mM of proline effectively preserved sperm progressive motility (*p* < 0.001) (Effect size = 0.034), the same effect was not detected in another treated group (4 mM) (*p* > 0.05) (Fig. 2-A). Furthermore, cryopreservation significantly impaired sperm viability (*p* < 0.001). The addition of 2 mM of proline helped maintain sperm viability compared to the control group (*p* < 0.001) (Effect size = 0.278). It is worth noting that increasing the proline concentration beyond 2 mM offered no notable benefits, as viability did not differ significantly from the control (*p* > 0.05) (Fig. 2-B).

### The effects of L-proline on the nitro-oxidative status of sperm medium

**Fig. 3:**
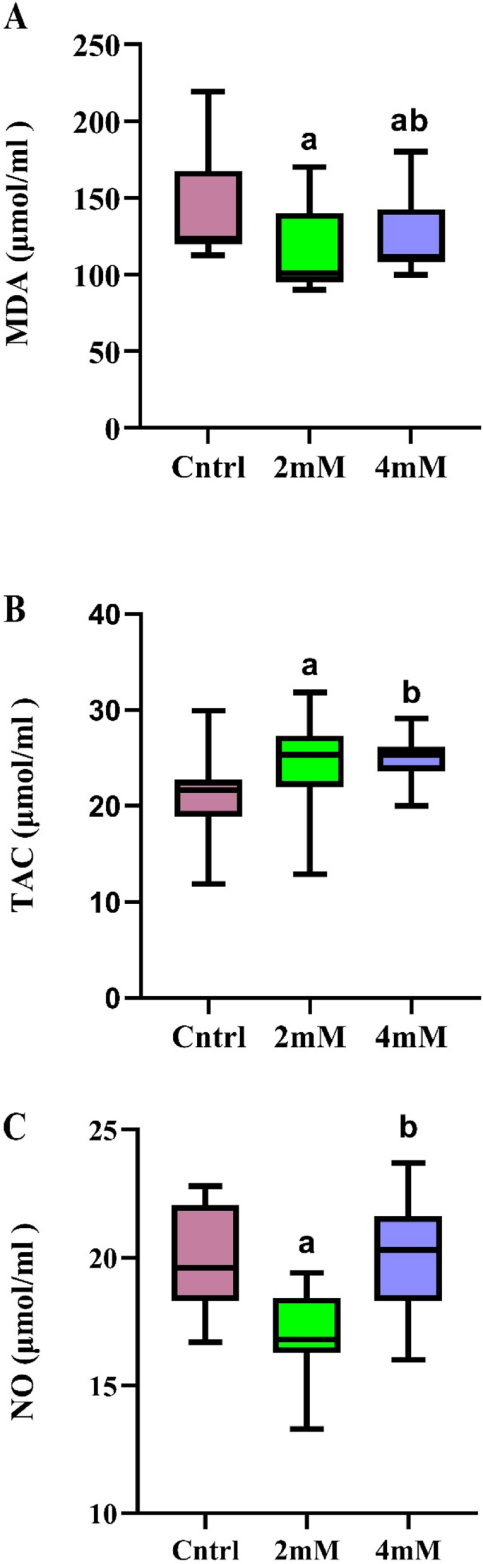
The effect of different concentrations of L-proline on MDA, and TAC and NO levels during cryopreservation (*n* = 30), presented as box-and-whisker plots showing median, interquartile range, and min/max values. **a** *p* < 0.05 versus the control group;**b** *p* < 0.05 versus 2 mM group

Figure 3 provides an overview of the LPO, demonstrating a significant reduction in MDA levels when 2 mM of proline was added compared to the control (*p* < 0.001) (Effect size = 0.156). Interestingly, even though the inclusion of 4 mM was able to diminish MDA levels compared to the control group (*p* < 0.001), the difference between 2 and 4 mM remained statistically significant (*p* < 0.001) (Fig. 3-A).

Our results demonstrated a significant increase in TAC in the group supplemented with 2 mM proline compared to the control group (*p* < 0.001) (Effect size = 0.185). While the 4 mM concentration also slightly elevated TAC levels, the increase was not statistically significant relative to the control (*p* > 0.05). Notably, the difference between the 2 mM and 4 mM groups was significant (*p* = 0.001), highlighting that 2 mM is the more effective concentration for maintaining TAC levels during the cryopreservation process (Fig. 3-B).

The findings demonstrated a significant reduction in NO levels with the inclusion of 2 mM proline (*p* < 0.001) (Effect size = 0.402), highlighting proline’s potential in mitigating NO-related oxidative stress. Interestingly, increasing the proline concentration to 4 mM did not sustain this effect, as NO levels returned to those observed in the control group. This suggests that while 2 mM proline effectively reduces NO levels, higher concentrations may not yield further benefits in NO reduction (Fig. 3-C).

### The effects of L-proline on sperm chromatin integrity

**Fig. 4:**
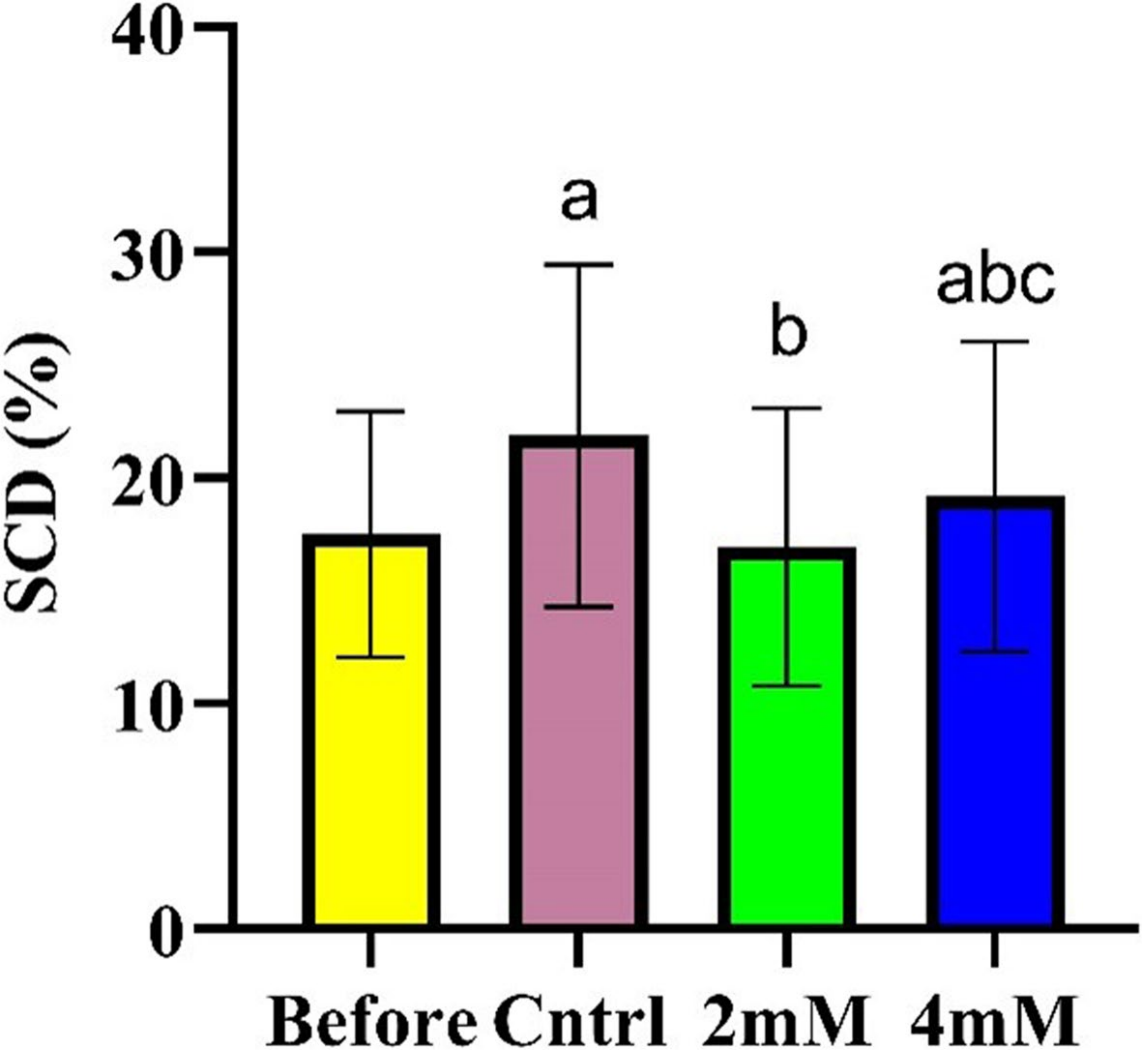
The effect of different concentrations of L-proline on SCD + spermatozoa during cryopreservation (*n* = 30), presented as mean ± SD. **a** *p* < 0.05 versus before cryopreservation; **b** *p* < 0.05 versus the control group;(c) *p* < 0.05 versus 2 mM group

**Fig. 5:**
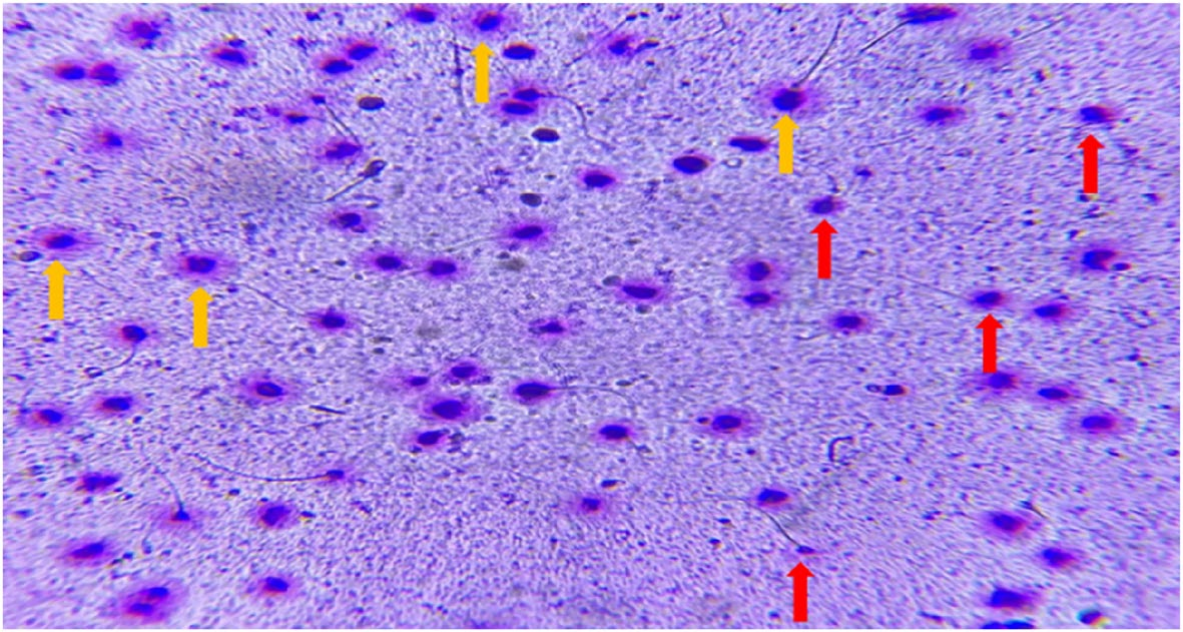
Representative image of sperm chromatin dispersion (SCD) staining. The yellow arrow indicates a normal sperm (large or medium halo), and the red arrow indicates an abnormal sperm (small or no halo)

In terms of sperm chromatic packaging and quality, freezing sperm adversely impacted chromatin integrity (*p* < 0.001). The addition of 2 mM proline significantly preserved sperm chromatin integrity during cryopreservation, restoring levels nearly to those observed prior to freezing (*p* < 0.001) (Effect size = 0.081). Although 4 mM proline also effectively contributed to maintaining chromatin quality (*p* < 0.028), it was comparatively less effective than the 2 mM concentration (*p* < 0.004). Notably, the difference in chromatin preservation between the 2 mM and 4 mM proline treatments was statistically significant (*p* < *0.004*), underscoring the superior efficacy of the 2 mM concentration in preserving chromatin integrity during cryopreservation (Figs. 4 and 5).

## Discussion

Proline has emerged as a promising agent due to its multifaceted protective roles in preserving sperm function during cryopreservation and incubation, as evidenced by studies in both animals and humans [15, 17, 22]. This study provides the first evidence that supplementation of sperm freezing media with 2 mM proline significantly enhances post-thaw sperm quality in asthenozoospermic samples. Notably, proline improved key functional parameters, progressive motility, viability, and chromatin integrity following cryopreservation. These improvements were associated with a marked reduction in nitro-oxidative stress markers, suggesting that the protective effects of proline are mediated through its antioxidative and osmoprotective properties.

Cryopreservation is widely recognized for its adverse effects on sperm parameters, particularly motility and viability [23]. Consistent with this, our study found that freezing significantly reduced progressive motility and viability of spermatozoa. However, the addition of 2 mM proline to the freezing medium effectively preserved these parameters, while a higher concentration (4 mM) provided no benefits on these parameters. These findings align with previous studies that highlight the dose-dependent effects of antioxidants. Optimal concentrations maximize protection against oxidative stress, but excessive levels may exert pro-oxidant effects and potentially harm cells [24, 25]. Notably, earlier research has demonstrated that high concentrations of proline and other amino acids, such as glutamine, can be toxic to spermatozoa in humans and animals [15, 26]. The protective effects of proline observed in our study can be attributed to its ability to stabilize mitochondrial function and neutralize ROS, both of which are essential for sustaining energy production required for motility [27]. In support of these observations, previous studies have demonstrated that proline supplementation enhances post-thaw motility and viability in both ram and bull spermatozoa [27, 28]. Additionally, a recent research underscores the role of proline as an osmolyte, contributing to the maintenance of cellular volume and membrane integrity during freeze–thaw cycles [29].

Oxidative stress arises from an imbalance between ROS and antioxidants, leading to structural and functional damage to spermatozoa [30, 31]. This includes compromised DNA integrity, impaired motility, and disrupted plasma membrane functionality, all of which are critical determinants of male fertility [32]. Our findings revealed that cryopreservation significantly elevated MDA levels in the sperm medium, a marker of LPO commonly used to assess oxidative damage. LPO plays a pivotal role in reducing sperm quality and function, ultimately contributing to male infertility [33]. Importantly, supplementing the freezing medium with 2 and 4 mM of proline significantly reduced MDA levels [34]. The observed reduction in LPO highlights proline’s ability to mitigate oxidative damage to the sperm membrane, a key factor in preserving sperm quality during cryopreservation [35]. The effect of proline on MDA levels is particularly significant. LPO of polyunsaturated fatty acids disrupts membrane fluidity and impairs the activity of membrane enzymes and ion channels, which ultimately hampers sperm motility [13]. Recent research further demonstrates that proline effectively penetrates cell membranes, scavenging intracellular ROS and inhibiting their formation, thereby reducing LPO [13, 35]. Additionally, proline forms a protective coating over the sperm surface through electrostatic interactions with the phosphate groups of plasma membrane phospholipids. This coating helps shield the sperm from damage caused by free radicals. Moreover, proline interacts with the phospholipid bilayer of the sperm membrane, helping to stabilize its structure and function. This stabilization enhances overall sperm resilience during cryopreservation [13, 15].

TAC reflects the concentration and efficiency of antioxidants in neutralizing free radicals within a sample and is commonly used as an indicator in male infertility studies [36]. Lower TAC levels in seminal plasma are frequently observed in infertile men, highlighting the role of oxidative stress in impaired sperm function [37]. In our study, TAC levels increased significantly with proline supplementation, reaching their peak at 2 mM. This result underscores the role of proline in enhancing the antioxidant defense system, either by directly scavenging ROS or by modulating endogenous antioxidant pathways [13, 27]. Proline's ability to mitigate oxidative stress during the freeze–thaw process has been positively correlated with improvements in key sperm parameters, including motility and viability [13]. Previous research further supports these findings, with reports indicating that proline not only scavenges ROS but also stabilizes cellular redox balance [35]. Liu et al. (2021) similarly demonstrated that proline enhances the activity of both enzymatic and non-enzymatic antioxidants, providing a robust defense against oxidative damage [38]. Consistent with these observations, our previous research also demonstrated that supplementation with 2 mM of proline significantly enhanced TAC levels in human sperm during 24 h of incubation [15].

While low concentrations of NO are known to enhance sperm motility and play a vital role in sperm capacitation, moderate to high levels can be harmful. These higher levels may reduce motility, impair mitochondrial respiration, and cause sperm toxicity [39, 40]. In our study, the freeze–thaw process led to a spike in NO levels, which correlated with the lowest observed sperm progressive motility. Elevated NO concentrations are known to inhibit mitochondrial respiration and cause DNA damage, further compromising sperm function [39, 41]. Interestingly, the addition of 2 mM proline significantly modulated NO levels. This highlights its potential in mitigating nitrosative stress, a critical contributor to sperm dysfunction [41]. These findings align with the studies demonstrating that reducing high levels of NO can enhance sperm quality [42, 43]. However, at a concentration of 4 mM proline, NO levels reverted to levels observed in the control group, potentially due to oxidative imbalances caused by excessive antioxidant activity acting as pro-oxidants [15]. Comparable reductions in oxidative and nitrosative stress markers, such as MDA and NO, have been reported with other antioxidants, including glutathione, α-tocopherol, and ascorbic acid [44]. Unlike these antioxidants, proline provides additional benefits by functioning as an osmolyte, stabilizing cellular volume, and enhancing membrane integrity, which further strengthens its protective role during cryopreservation [13, 35].

Men with normal fertility generally exhibit lower levels of sperm DNA damage, whereas men with fertility challenges often show higher levels of DNA fragmentation. Elevated sperm DNA fragmentation is associated with reduced natural fertility, recurrent pregnancy loss, and unexplained infertility [45]. Consistent with previous research, our findings demonstrated that cryopreservation significantly increased DNA fragmentation. This is likely due to oxidative damage caused by the freeze–thaw process, which promotes sperm DNA fragmentation and apoptosis [46]. This was further supported by observed peaks in MDA and NO levels after thawing, highlighting the role of nitro-oxidative stress in chromatin and DNA damage during cryopreservation [35]. SCD analysis in this study revealed that supplementation with 2 and 4 mM proline effectively preserved chromatin integrity during cryopreservation. These results align with previous findings and demonstrate the protective effects of proline on sperm chromatin under oxidative stress [16, 47]. This is particularly significant, as sperm chromatin integrity is a crucial factor in successful fertilization, embryo development, and pregnancy outcomes [48]. The ability of proline to maintain chromatin integrity can be attributed to its role as an osmolyte, which stabilizes cellular structures and protects chromatin from ROS-induced damage [15, 22]. Accordingly, Chuang Liu et al. (2023) demonstrated that adding proline to a freezing extender improved chromatin integrity and protected sperm from cryopreservation-induced apoptosis in frozen-thawed boar semen [47]. Similarly, incorporating other antioxidants into cryopreservation media has been shown to enhance sperm parameters, improve chromatin quality, and positively influence subsequent IVF outcomes. This emphasizes the critical role of antioxidant supplementation in mitigating cryopreservation-induced damage [49].

Despite these promising findings, the study has several limitations. The primary limitation is its in vitro design, which focuses on laboratory parameters rather than clinical endpoints. While improvements in motility, viability, and chromatin integrity are encouraging, future research is essential to determine if these benefits translate to functional outcomes like fertilization capacity, embryo development, and pregnancy success rates. Additionally, while our findings point to the mitigation of nitro-oxidative stress, the specific molecular mechanisms underlying proline's protective effects were not fully elucidated. Therefore, larger-scale clinical trials are needed to validate these results and confirm the utility of proline supplementation in a clinical ART setting.

## Conclusion

This study highlights the potential of proline, particularly at a concentration of 2 mM, as a promising additive for enhancing sperm quality during cryopreservation in asthenozoospermic samples. By improving progressive motility, viability, and chromatin integrity while alleviating oxidative stress, proline supplementation offers a valuable approach to improving ART outcomes in men with asthenozoospermia. Future studies should explore the potential synergistic effects of combining proline with other antioxidants or cryoprotectants to further enhance post-thaw sperm quality.

## Data Availability

The datasets used and analysed during the current study are available from the corresponding author on reasonable request.
